# MHC class II presentation of FVIII-AnnexinA5 fusion proteins internalized by antigen presenting cells

**DOI:** 10.3389/fimmu.2025.1668397

**Published:** 2025-09-25

**Authors:** Mariarosaria Miranda, Michela Leoni, Carmen van der Zwaan, Robin van Bruggen, Chris P. M. Reutelingsperger, Karin Fijnvandraat, Arie J. Hoogendijk, Maartje van den Biggelaar, Jan Voorberg

**Affiliations:** ^1^ Department of Molecular Hematology, Sanquin Research and Landsteiner Laboratory, Amsterdam, Netherlands; ^2^ Department of Biochemistry, Cardiovascular Research Institute Maastricht, Maastricht University, Maastricht, Netherlands; ^3^ Department of Pediatric Hematology, Amsterdam University Medical Center (UMC), University of Amsterdam, Emma Children’s Hospital, Amsterdam, Netherlands; ^4^ Department of Experimental Vascular Medicine, Amsterdam UMC location University of Amsterdam, Amsterdam, Netherlands

**Keywords:** FVIII, annexin A5, antigen presentation, phosphatidylserine, red blood cells

## Abstract

**Introduction:**

The development of neutralizing antibodies (inhibitors) against coagulation factor VIII (FVIII) remains the most serious complication in the treatment of hemophilia A. While immune tolerance induction (ITI) is the standard strategy to eliminate these antibodies, it fails in approximately 30% of patients with severe hemophilia A, underscoring the need for innovative approaches to promote FVIII-specific tolerance.

**Methods:**

To address this challenge, we generated fusion proteins composed of A2, A3-C1-C2 (light chain, LCh), and C2 domains of FVIII linked to Annexin A5 (AnxA5), a protein that binds phosphatidylserine (PS), a hallmark of apoptotic cells.

**Results:**

ELISA confirmed high-affinity binding of all fusion proteins to immobilized PS. To model PS exposure *in vitro*, red blood cells (RBCs) were treated with phorbol 12-myristate 13-acetate (PMA), leading to the release of PS-exposing microvesicles. Flow cytometry showed that FVIII-AnxA5 fusion proteins selectively bound to PS-exposing microvesicles but not to intact RBCs. Using mass spectrometry-based immunopeptidomics, we demonstrated that macrophages pulsed with FVIII-AnxA5 fusion proteins efficiently processed and presented FVIII-derived peptides on HLA-DR molecules.

**Conclusions:**

These findings suggest that FVIII-AnxA5 fusion proteins can engage apoptotic cell clearance pathways to facilitate antigen presentation in a potentially tolerogenic context. This strategy may offer a novel means of inducing immune tolerance to FVIII in hemophilia A.

## Introduction

Hemophilia A is a bleeding disorder caused by a mutation in the *F8* gene, resulting in a deficiency of functional clotting factor VIII (FVIII) (1). The deficiency or dysfunction of FVIII impairs the formation of a stable fibrin clot, leading to prolonged bleeding episodes. The cornerstone of hemophilia A treatment is replacement therapy, which involves the administration of FVIII to restore normal hemostasis (1). Over the past decade, bioengineered FVIII molecules with extended half-life (EHL) have been developed to reduce the burden of frequent intravenous injections, improving patient adherence and quality of life (2–4). Despite these advancements, the primary complication of replacement therapy remains the development of anti-FVIII antibodies (so called FVIII inhibitors), which occur in 25-40% of patients with severe hemophilia A (5, 6). FVIII inhibitors significantly reduce the efficacy of FVIII infusions thereby severely complicating managements of bleeds (7). To overcome this challenge, bypassing agents such as activated prothrombin complex concentrate (aPCC) and recombinant activated factor VII (rFVIIa) have been employed to restore hemostasis through FVIII-independent pathways (8, 9). Non-factor replacement therapies have emerged as effective prophylactic options. Emicizumab, a bispecific monoclonal antibody that bridges activated factor IX and factor X, functionally mimics FVIII and even secure hemostasis in patients with high-titer inhibitors (10–13). Building on its excellent clinical profile in inhibitor patients as well as its convenient relative infrequent subcutaneous administration emicizumab is now widely used for prevention of bleeds in patients with hemophilia A (14, 15). While emicizumab is effective for routine prophylaxis, FVIII infusions remain necessary to treat breakthrough bleeding or to prevent bleeding during surgery (16). It is currently not clear whether infrequent exposure to FVIII puts these patients at risk of inhibitor development (10). Currently there are limited clinical protocols available for restoring tolerance in patients with hemophilia A; immune tolerance induction (ITI) remains the only established strategy to eradicate FVIII-specific antibodies. ITI involves high-dose, repeated FVIII infusions to induce a state of peripheral immune tolerance (17). Although effective in many cases, ITI is a costly and time-consuming treatment that requires frequent infusions and fails in approximately 30% of patients (18, 19). Clearly, there is a need for alternative tolerization strategies to protect patients receiving emicizumab, or other non-factor replacement therapies, from the risk of inhibitor development following breakthrough bleeds or surgical interventions (6).

FVIII inhibitory antibodies predominantly belong to the IgG subclasses, with IgG4 and IgG1 being the most abundant (20–22). This pattern reflects the role of CD4^+^ T cells in driving the FVIII inhibitor response. A recent study by Kaczmarek et al. revealed that FVIII is recognized by marginal zone B cells and marginal metallophilic macrophages (23). These antigen presenting cells (APCs) transport FVIII to the white pulp, where conventional dendritic cells (cDCs) prime helper T cells, which subsequently differentiate into follicular helper T (Tfh) cells. This process promotes T-cell proliferation and antibody production. In contrast, a less immunogenic protein, such as chicken ovalbumin (OVA), is taken up primarily by red pulp macrophages (RPMs) and does not trigger the development of inhibitory antibodies (23).

Under quiescent conditions, approximately one million cells per second die through apoptosis in the human body (24). The engulfing of dead cells by professional phagocytes, known as efferocytosis, is essential to clear apoptotic cells and maintain homeostasis (24–26). Efferocytosis occurs in all major tissues and organs, ensuring that processes such as removal of aged neutrophils and red blood cells (RBCs) and clearance of negatively selected thymocytes are executed rapidly (27). These clearance processes are performed by professional phagocytes, such as macrophages and dendritic cells (DCs), and non-professional phagocytes, such as fibroblast and epithelial cells (24). Apoptotic cells expose ‘eat me’ signals on their surface, which are sensed by phagocytes through their receptors and bridging molecules (28–30). The main cell surface receptors involved are the low-density lipoprotein receptor-related protein 1, T cell immunoglobulin mucin receptor (TIM) 1, TIM3, TIM4, adhesion G protein-coupled receptor B1, stabilin-1 and stabilin-2, while among the main bridging molecules there are protein S, milk fat globule epidermal growth factor 8 (MFG-E8) and vitamin K-dependent protein growth arrest specific 6 (Gas6) (24, 31). The most well characterized “eat-me” signal is phosphatidylserine (PS), which is found in the inner leaflet of living cells and is exposed externally via the action of caspase-regulated flippase during apoptosis (32, 33). PS is recognized directly by PS binding receptors (e.g. stabilin-1, stabilin-2, TIM4) or indirectly by bridging mediators (31).

A crucial aspect of efferocytosis is the clearance of senescent erythrocytes, a process essential for erythropoietic homeostasis and systemic iron recycling (27). Removal of senescent RBC from the circulation occurs through phagocytosis, which takes place mainly in macrophages of the spleen, but also in the liver and the bone marrow (34). Red pulp macrophages (RPMs) in the spleen and Kupffer cells (KC) in the liver are specialized in recognizing and engulfing aged RBCs (34, 35). Under physiological conditions, RBCs express CD47, which interacts with signal regulatory protein α (SIRP-α) on RPMs and KCs, delivering a ‘don’t eat me’ signal that prevents premature phagocytosis (36, 37). As RBCs age, they progressively accumulate removal signals including conformational changes in CD47, oxidation of proteins and lipids, loss of membrane deformability, activation of adhesion molecules, and PS exposure (38). PS is recognized by a specialized set of receptors predominantly expressed on RPMs and liver macrophages, including TIM4, CD36, and TAM receptors (39, 40). Following engulfment, the enzyme heme oxygenase-1 (HMOX-1) catalyzes the breakdown of heme thereby promoting iron recycling and protecting tissues from heme-induced toxicity (35, 41). Furthermore, RPMs are pivotal in maintaing immune tolerance, as their ability to phagocytose self-antigens leads to the deletion of antigen-specific T cells (35, 42). This function is enabled by their expression of MHC class I and II molecules, alongside the absence of costimulatory molecules like CD80 and CD86 (35). RPMs also promote the secretion of anti-inflammatory cytokines, such as IL-10 and TGF-β, and enhance Foxp3 expression in CD4+ T cells, thereby fostering the generation of regulatory T cells (35, 42). Similarly, KCs exert their tolerogenic activity, which is crucial for preventing undesired immune responses under physiological conditions, by suppressing T cell activation through the production of prostaglandin E2 (PGE2), 5-deoxy-delta12,14-PGJ2 (15d-PGJ2), IL-10, and TGF-β (43–46).

In this study, we designed fusion proteins by combining FVIII domains with Annexin A5 to specifically target PS exposing apoptotic cells. We evaluated the binding of FVIII-AnxA5 fusion proteins to RBC derived PS exposing microvesicles. In parallel we also assessed MHC class II presentation of FVIII-AnxA5 fusion proteins by macrophages. Our findings suggest that Annexin A5-fused antigens can engage apoptotic pathways to modulate immune responses, highlighting their potential for immune regulation.

## Materials and methods

### Subjects

Blood was drawn from healthy volunteers in accordance with Dutch regulations and following approval from Sanquin Ethical Advisory Board in accordance with the Declaration of Helsinki. Peripheral blood mononuclear cells (PBMC) were isolated from freshly drawn, EDTA anticoagulated blood by separation over a Ficoll-Paque TM PLUS gradient (GE Healthcare). Red blood cells (RBCs) were isolated from freshly drawn, citrate anticoagulated blood by centrifugation at 105 g for 5 minutes (Eppendorf 5417R Refrigerated Centrifuge). After removing the platelet-rich plasma (PRP) and PBMC, erythrocytes were washed twice with saline-adenine-glucose-mannitol (SAG-M; 150 mM NaCl, 1.25 mM adenine, 50 mM glucose, 29 mM mannitol; Compoflex^®^, Fresenius Kabi) and resuspended in SAG-M. Final concentration of RBCs was determined with an Advia 2120 (Siemens Medical Solutions Diagnostics).

### Reagents

Annexin AnxA5, A2- AnxA5, C2- AnxA5 and A3-C1-C2 (light chain, LCh)- AnxA5 encoding cDNAs were ordered from GENEWIZ/Azenta, StrepTrap XT 1mL prepacked columns from Cytiva, Brain PS L-α-phosphatidylserine from Avanti Polar Lipids, anti-V5-HRP Antibody from Invitrogen, CD14 microbeads, and manual MACS Magnetic Separators for cell separation from Miltenyi; M-CSF from Peprotech; Human Serum Albumin (HSA) (200 g/l) from Sanquin; ExpiCho Expression System, IMDM, RPMI 1640, UltraPure™ 0.5M EDTA (pH 8.0), Alexa Fluor 555 labeling kit, Alexa Fluor 647 labeling kit, Vybrant™ DiD Cell-Labeling Solution, and Vybrant™ DiO Cell-Labeling Solution from ThermoFisher. InVivoMAb anti-human/monkey HLA-DR (L243) was obtained from BioXCell. Purified monoclonal antibody L243 was coupled to CNBr Sepharose 4B at a final concentration of 2 mg/ml. Phorbol 12-myristate 13-acetate (PMA), L-glutamine and Penicillin Streptomycin Solution, Pen-Strep (10,000 units penicillin and 10 mg streptomycin/mL) from Sigma.

### FVIII-AnxA5 fusion proteins design and expression

Five constructs were designed and synthesized: three FVIII-Annexin V (AnxA5) fusion proteins (A2-AnxA5, C2-AnxA5, and LCh-AnxA5), AnxA5 alone, and the FVIII light chain (LCh) alone. All constructs were cloned into the pcDNA3.1(+) vector. Each fusion protein included an N-terminal mouse Igκ-chain signal peptide (METDTLLLWVLLLWVPGSTGD) to promote secretion (47). The synthetic FVIII-derived regions included the A2 domain (Ser392–Pro758), the C2 domain (Ser2192–Tyr2350), and the light chain (LCh, Glu1668–Tyr2350) were ordered at Genewiz and inserted into the pcDNA3.1(+) construct using AgeI and XhoI. These domains were linked via a flexible (GGGGS)_3_ GS linker to human AnnexinA5 (UniProt ID: P08758). A C-terminal V5 epitope tag (GKPIPNPLLGLDST) and Twin-Strep tag (WSHPQFEKGGGSGGGSGGSAWSHPQFEK) were included in the constructs to allow for protein detection and purification. Proteins were expressed in ExpiCHO using the High Titer expression protocol with 1 μg of DNA per mL of cell culture as recommended by the manufacturer. Five days after transfection cell culture supernatant was collected by centrifugation at 4000g for 30 minutes at 4°C. A final concentration of 10 mM benzamidine was added to the supernatant and aliquots were stored at −30 °C until use. Expression was confirmed by Western blot using anti-V5-horseradish peroxidase (HRP) (1:15000; Invitrogen, R961-25). The proteins were purified from the media using StrepTrap XT prepacked column in the ÄKTA pure™ chromatography system (Cytiva). The proteins were washed with 100mM Tris-HCl, 150mM NaCl, pH 8.0, and eluted in 100mM Tris-HCl, 150mM NaCl, 5mM CaCl_2,_ 100mM biotin, pH 8.0; the buffer was exchanged to 25mM Hepes, 150mM NaCl, 2mM CaCl_2_, pH 7.4 by dialysis overnight. The success of the purification was assessed by SDS page gel and Coomassie brilliant blue (CBB) staining. The concentration of the protein was determined by Bradford assay using the Protein Assay kit (Bio-Rad), following the manufacturer’s instructions. The proteins were aliquoted, snap-frozen and kept at -80°C until use.

### FVIII-AnxA5 fusion proteins interaction with PS

Polysorp microtiter plates (Nunc) were coated with PS (2 µg/mL) in ethanol and incubated overnight at room temperature until complete evaporation. The plates were then blocked with blocking buffer (10 mM Tris-HCl, pH 7.4, 150 mM NaCl, 3% BSA) for 1 hour at room temperature. Following blocking, each of the four AnxA5 fusion proteins was individually diluted in sample buffer (10 mM Tris-HCl pH 7.4, 150 mM NaCl, 5 mM CaCl_2_) and added to separate wells, then incubated for 1 hour at room temperature. After incubation, anti-V5-HRP detection antibody (1:2000 dilution in sample buffer) was added and incubated for 1 hour at room temperature. For detection, the plate was developed with TMB substrate solution (0.1 mg/mL TMB in DMSO, 0.11 M sodium acetate, pH 5.5, 0.0045% H_2_O_2_) for 10 minutes. The reaction was then stopped by adding 100 μl of 1 M H_2_SO_4_. Optical density (OD) was read at 450 nm with a 540 nm reference on a SpectraMax Plus plate reader. Data were analyzed with GraphPad Prism v10.3 (GraphPad Software). Data were fitted by nonlinear regression to a four-parameter logistic (4PL) sigmoidal dose–response model (variable slope) to determine EC_50_ values.

### FVIII-AnxA5 fusion proteins interaction with RBC

To investigate the interaction between FVIII-AnxA5 fusion proteins and phosphatidylserine (PS) on RBCs, we induced PS exposure on RBCs. A total of 0.5x10^6^ RBCs were incubated with phorbol 12-myristate 13-acetate (PMA) at a final concentration of 6 μM. The incubation was performed in 100 μl of buffer A (20 mM HEPES pH 7.4, 132 mM NaCl, 6 mM KCl, 1 mM MgSO_4_, 1.2 mM K_2_HPO_4_, 1 mM CaCl_2_, 1% glucose supplemented with 0.5% HSA) for 30 minutes at 37°C. AnxA5 and FVIII-AnxA5 constructs were pre-labelled with Alexa Fluor 555 and Alexa Fluor 647, respectively, following the manufacturer’s protocol. Protein concentrations and dye-to-protein ratios (DOL) were determined spectrophotometrically according to the kit instructions. Upon PMA incubation, RBCs were washed twice with buffer A and incubated with either pre-labelled AnxA5 and FVIII-AnxA5 fusion proteins in a concentration range from 0.01 to 100 nM in buffer B (20 mM HEPES pH 7.4, 132 mM NaCl, 6 mM KCl, 1 mM MgSO_4_, 1.2 mM K_2_HPO_4_, 2.5 mM CaCl_2_, 1% glucose supplemented with 0.5% HSA) for 30 min at RT. Cells were washed and analyzed by flow cytometry (BD LSR II Flow Cytometer).

### FVIII-AnxA5 fusion proteins loaded on macrophages

Monocytes were isolated from the peripheral blood mononuclear cell fraction by positive selection using CD14 microbeads and a magnetic cell separator. Monocytes were plated at a concentration of 1x10^6^ cells in a total volume of 2 ml in a 6-well plate (Nunc, Roskilde Denmark) in IMDM complete (2 mM L-glutamine, penicillin–streptomycin 10 U/mL, and 10% fetal calf serum), and supplemented with 50 ng/ml M-CSF for 10 days. At day 3 and day 7, half of the medium was refreshed with IMDM supplemented with M-CSF (50 ng/ml). Upon 10 days of culture, 1x10^6^ macrophages were washed and incubated with 100 nM of each of the four AnxA5 fusion proteins overnight in a final volume of 2 ml IMDM complete.

### Phagocytosis of PMA treated RBC preloaded by FVIII-AnxA5 fusion by macrophages

Monocytes were isolated and macrophages were differentiated as previously mentioned. Upon 10 days of culture, RBCs were isolated, treated with PMA, and incubated with AnxA5 and FVIII-AnxA5 constructs as previously described. In total 1x10^6^ macrophages were washed and incubated with FVIII-AnxA5 pre-loaded RBCs overnight in a final volume of 2 ml IMDM complete. The ratio of macrophages and red blood cells was 1:100.

### Purification of HLA-DR presented peptides on macrophages

FVIII-AnxA5 and FVIII-AnxA5-RBCs-loaded monocyte derived macrophages were harvested and resuspended in 500 μL of lysis buffer (10 mM Tris-HCl pH 8.0, 0.25% octyl-β-D-glucopyranoside, 1% sodium deoxycholate and 10 mM EDTA) for 30 min at 4°C. Lysates were then centrifuged at 20,000 x g for 15 min at 4°C and supernatants were incubated with 300 μL of Sepharose beads containing 2 mg/ml of anti-HLA-DR monoclonal antibody L243 as described previously (48). Following overnight incubation at 4°C, L243-containing Sepharose beads were washed twice with lysis buffer, and five times with 10 mM Tris-HCl pH 8.0. Bound MHCII molecules were eluted by incubation with 10% acetic acid for 10 min at room temperature. Eluates were collected and heated for 15 min at 70°C to dissociate peptide/MHCII complexes.

### Mass spectrometry

Samples were desalted using Empore C18 STAGE tips which were prepared in-house. STAGE tips were equilibrated with 100% acetonitrile and subsequently washed with 1% formic acid. Samples were loaded on Empore C18 STAGE tips and washed once with 1% formic acid and once with 1% formic acid supplemented with 5% acetonitrile. Peptides were eluted from Empore C18 STAGE tips with 60 μL 1% formic acid supplemented with 30% acetonitrile and concentrated to a final volume of 5 μL using vacuum centrifugation. Eluted peptides were separated using columns (New Objective type FS360-75-8-N-5-C20, Inc., Woburn, MA, USA) filled with 1.9 μm C18 particles (Dr.Maisch, Ammerbuch-Entringen, Germany) at a flow rate of 300 nL/min, HPLC Buffer A was composed of 0.1% formic acid in water and buffer B of 0.1% formic acid, 80% acetonitrile. Peptides were loaded for 17 min at 300 nL/min at 5% buffer B, equilibrated for 5 minutes at 5% buffer B (17–22 min) and eluted by increasing buffer B from 5-10% (22–27 min), 10-28% (27–70 min) and 28-85% (70–84 min) followed by a 6 minute wash to 95% and in 1 minute ramped to 5%, follow by a 4 min equilibration at 5%. Column eluate was sprayed directly into the Orbitrap Fusion Lumos Tribrid mass spectrometer (Thermo Fisher Scientific Inc., Bremen, Germany) using a nano electrospray source with a spray voltage of 2.15 kV. Survey scans of peptide precursors from 300 to 1600 m/z were performed at 120K resolution (at 200 m/z) in profile mode with a AGC target of 250% and a maximum injection time of 50 ms. Only precursors with charge state 2–7 were sampled for MS2. The dynamic exclusion duration was set to 20 s with a 10 ppm tolerance around the selected precursor and its isotopes. Monoisotopic precursor selection was turned on and an intensity threshold of 2e4 was set. The instrument was run in top speed mode with 3 s cycles. Tandem mass spectrometry (MS/MS) was performed using a quadrupole mass analyzer for ion isolation and higher-energy collisional dissociation (HCD) for fragmentation. The isolation window was set to 0.7 Da, and the normalized collision energy for HCD was 30. Fragment ions were analyzed in the Orbitrap at a resolution of 60k in centroid mode with a mass range of 200–1400 m/z, AGC target of 300% and a maximum injection time of 118 ms. All data were acquired with SII for Xcalibur software.

### Data analysis flow cytometry and mass spectrometry

Flow cytometry data were analyzed with FlowJo (BD Biosciences). Peptides were identified using Proteome Discoverer 2.2 (Thermo Scientific, Bremen, Germany). Mass spectrometry raw data (Xcalibur format) were analyzed using both Proteome Discoverer 2.2 (Thermo Scientific, Bremen, Germany) and the FragPipe pipeline (v20.0) incorporating MSFragger (v3.8). For both platforms, the following search parameters were applied: precursor mass tolerance of ±10 ppm, fragment mass tolerance of 0.02 Da, peptide mass range of 3000–7000 Da, peptide length between 6 and 50 amino acids, and a false discovery rate (FDR) threshold of 0.05. In Proteome Discoverer, data were searched against the UniProtKB *H. sapiens* reviewed database (downloaded 2023), supplemented with FVIII-AnxA5 fusion protein sequences. Only peptides with medium or high confidence were retained and grouped by donor. In FragPipe, searches were performed against the human SwissProt database (release 2021.22.04), supplemented with decoy sequences (total 40,887 entries, 50% decoys). Digestion was set to non-specific to allow detection of peptides within the defined mass and length constraints. Peptide quantification was performed using IonQuant (v1.9.8) under default parameters, with a 10 ppm mass tolerance and match-between-runs disabled.

## Results

### Binding of FVIII-AnxA5 fusion proteins to PS

**Figure 1:**
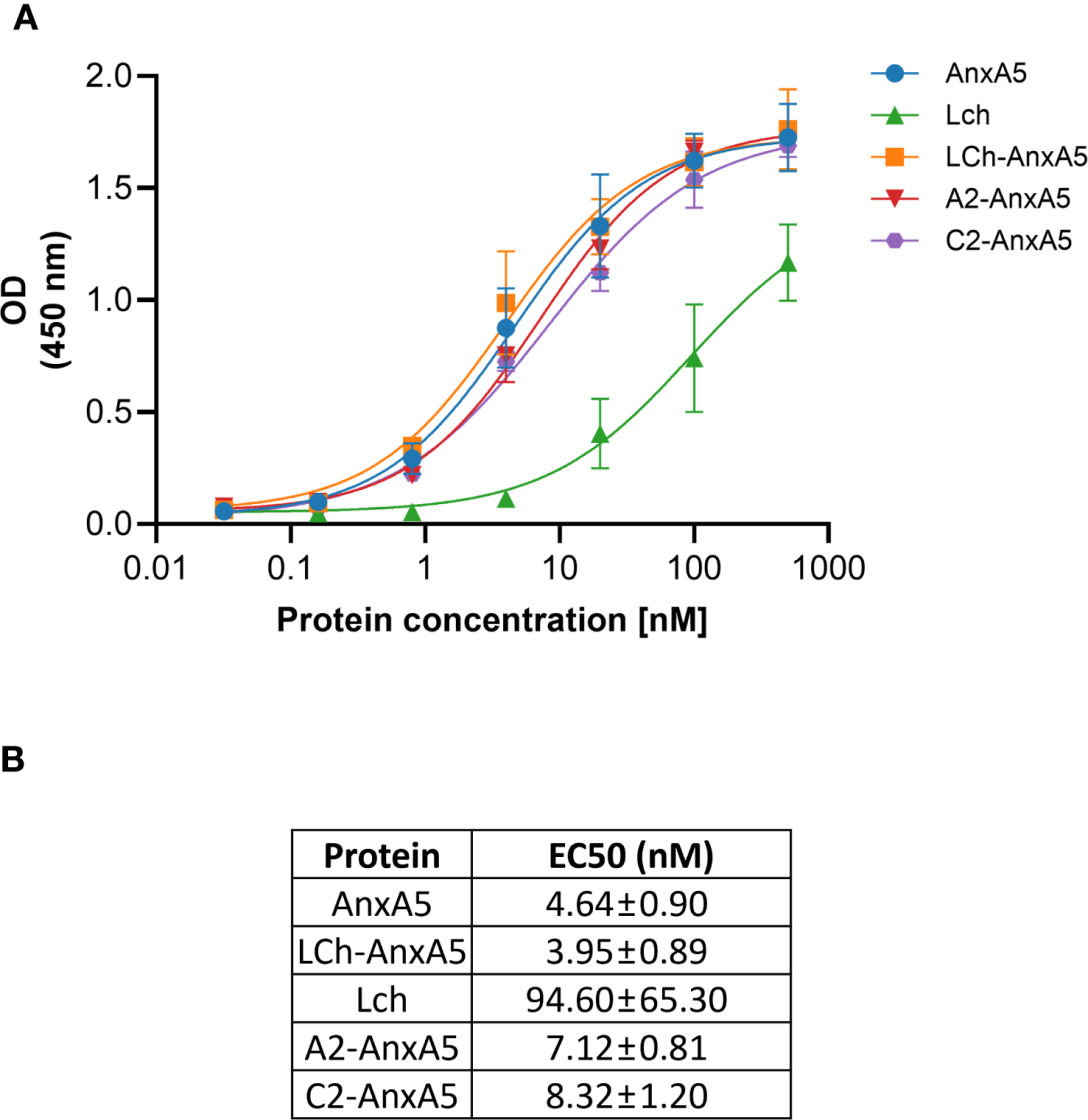
Dose-response binding of FVIII-AnxA5 fusion proteins to phosphatidylserine (PS). Polysorp microtiter plates were coated with PS and incubated with increasing concentrations (nM) of five different proteins: AnxA5 alone, LCh FVIII, and three FVIII- AnxA5 fusion constructs (LCh- AnxA5, A2- AnxA5, C2- AnxA5). Binding was detected using anti-V5-HRP antibody, and optical density (OD) was measured at 450 nm. All proteins exhibited dose-dependent binding to PS with similar affinities, except for LCh FVIII, which showed reduced binding consistent with the absence of Annexin A5. The binding of LCh FVIII to PS is mediated by the C2 domain. Panel **(A)** shows the binding curves, and panel **(B)** displays the corresponding EC50 values, calculated by fitting the data to a 4 parameters sigmoidal dose response model.

To enable specific targeting of phosphatidylserine (PS) exposed on apoptotic cells, we generated a series of FVIII-AnxA5 fusion proteins. The design incorporated different FVIII domains, including the highly immunogenic A2, C1, and C2 domains, fused to Annexin A5, and the corresponding amino acid sequences are presented in **Supplementary Figure 1** (49). To evaluate the PS-targeting capability of the designed FVIII-AnxA5 fusion proteins, plates coated with PS were incubated with increasing concentrations of the three fusion constructs, alongside controls including AnxA5 alone and the FVIII light chain composed of the A3-C1-C2 domains of FVIII. As shown in **Figure 1**, all fusion proteins demonstrated a clear, sigmoidal dose-dependent binding to PS, exhibiting binding profiles similar to that of AnxA5. The FVIII light chain exhibited reduced binding to PS, consistent with the absence of the AnxA5; however, residual binding was still observed due to the PS-binding properties of the C2 domain (50, 51). Half maximal binding of LCh-AnxA5, A2-AnxA5 and C2-AnxA5 fusion proteins was observed at 40 times lower concentrations when compared to FVIII light chain. The corresponding EC50 values are reported in **Figure 1B**. For AnxA5 and the FVIII–AnxA5 fusion proteins, EC50 ranged from 3.92 to 8.32. In contrast, the FVIII light chain without Annexin A5 exhibited a substantially higher EC50 of 94.60 nM. The difference between AnxA5 containing proteins and FVIII LCh without Annexin A5 is consistent with published affinities values (52, 53). These findings show that FVIII-AnxA5 fusion proteins bind with higher affinity to PS when compared to FVIII. Overall, our data demonstrate that the FVIII-AnxA5 fusion proteins strongly bind to PS allowing for exploring their use to selectively target FVIII domains to PS exposing apoptotic cells.

### FVIII-AnxA5 fusion proteins interaction with PS-exposed red blood cells

**Figure 2:**
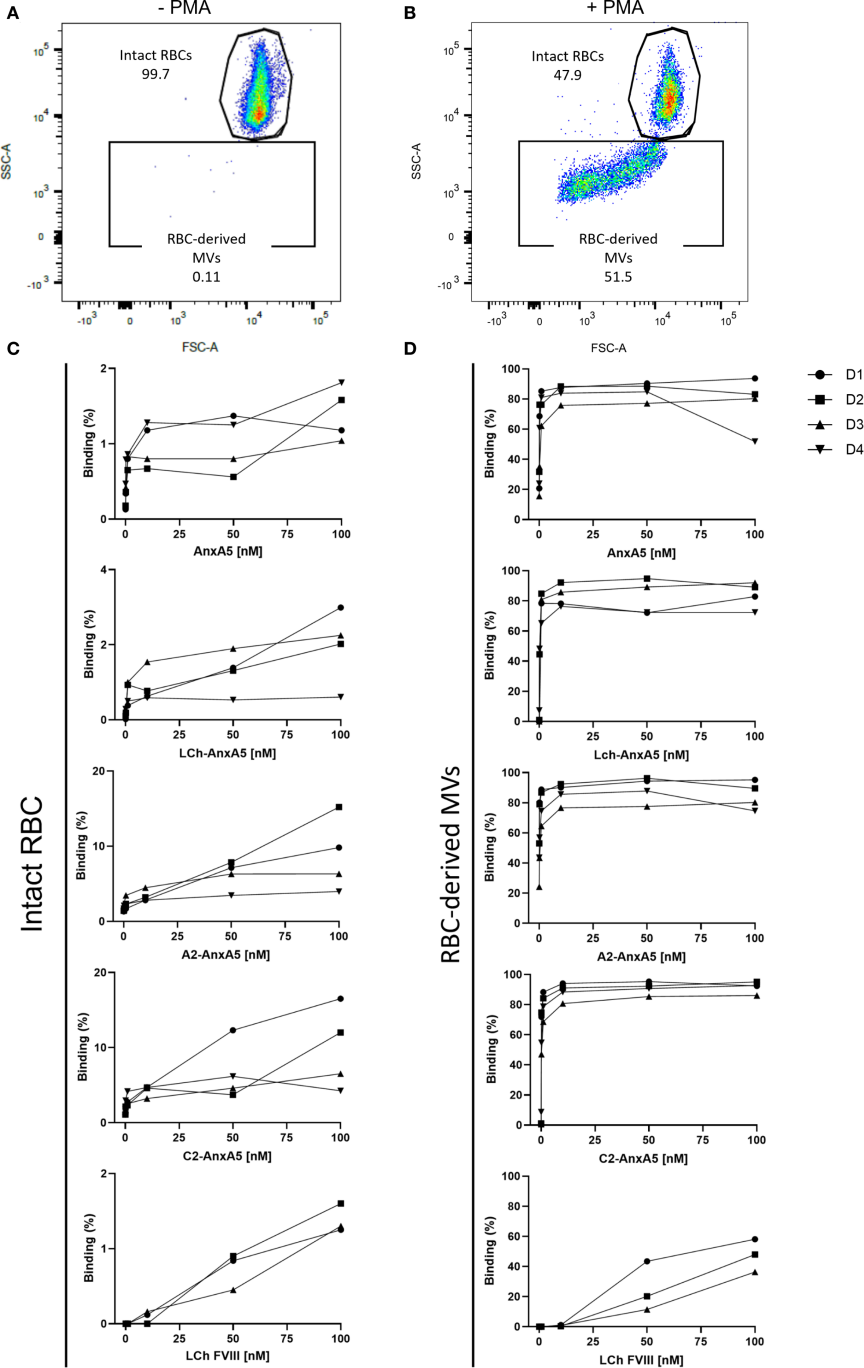
FVIII- AnxA5 fusion proteins interaction with PS exposed red blood cells. The interaction of FVIII-AnxA5 fusion proteins with RBCs was evaluated using flow cytometry. Untreated RBCs were gated according to the forward (FSC) and side (SSC) scatter parameters **(A)**. Upon treatment with PMA, RBC-derived microvesicles (MVs) with a smaller size emerged **(B)**. The binding of AnxA5 only, LCh- AnxA5, A2- AnxA5, C2- AnxA5, and LCh- AnxA5 to intact **(C)** and RBC-derived microvesicles (MVs) **(D)** was expressed as percentage of cells positive to the fusion protein signal. AnxA5 and all the FVIII- AnxA5 fusion proteins bound to vesiculating RBCs, while their binding to intact RBCs was minimal.

To evaluate the binding of FVIII–AnxA5 fusion proteins to PS-exposed red blood cells (RBCs), we performed flow cytometric analysis using RBCs isolated from multiple healthy donors. In untreated samples, RBCs were gated based on their characteristic forward and side scatter profiles (**Figure 2A**). Upon treatment with PMA, an additional population of RBC-derived microvesicles (MVs) emerged, as evidenced by altered forward and side scatter properties (**Figure 2B**). Intact RBCs and RBC-derived MVs were gated as separate populations, and the binding of fluorescently labeled FVIII-AnxA5 fusion proteins was assessed. To account for background fluorescence, a cut-off for FVIII-AnxA5-AF647 positivity was established using PMA-treated RBCs that were not incubated with the fusion protein. Events with fluorescence intensity exceeding this threshold were considered positive for binding of FVIII-AnxA5 fusion proteins. As a positive control, we first assessed the binding of AnxA5 alone to confirm PS exposure on RBC-derived MVs. Minimal fluorescence was detected on intact RBCs, indicating negligible PS exposure (**Figure 2C**). In contrast, a strong dose-dependent binding was observed for the RBC-derived MVs (**Figure 2D**). A similar binding pattern was observed for the LCh–AnxA5, A2–AnxA5, and C2–AnxA5 fusion proteins, all of which showed low binding to intact RBCs and robust binding to RBC-derived MVs (**Figure 2D**). Notably, the FVIII light chain (LCh) alone also showed low but detectable binding to RBC-derived MVs, consistent with its binding to immobilized PS, albeit to a lesser extent when compared to the FVIII-AnxA5 fusion proteins (**Figures 2C, D**). These results demonstrate that the FVIII–AnxA5 fusion proteins preferentially interact with RBC-derived MVs that expose PS.

### Phagocytosis of PS-exposed red blood cells by macrophages

**Figure 3:**
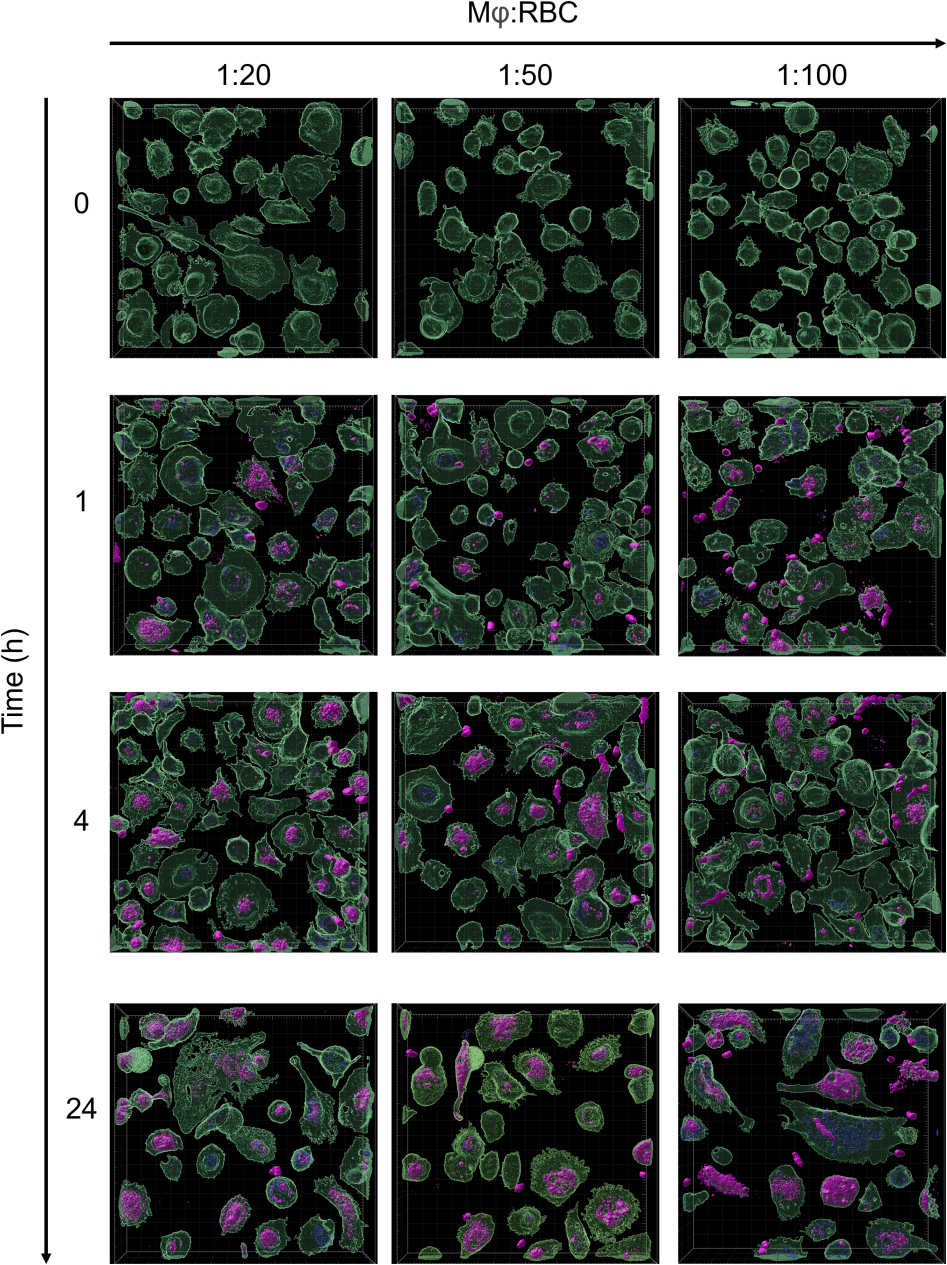
Phagocytosis of PS-exposing red blood cells by macrophages. RBCs were stained with Vibrand DiD (purple). Upon treatment with PMA, PS-exposing RBCs were loaded with AF555 labelled AnxA5 (blue). Vibrant DiO-labeled monocytes-derived macrophages (green) were incubated with AnxA5 loaded RBCs at Mφ:RBC ratios of 1:20, 1:50 and 1:100 for 0, 1, 4 and 24 hours. After removing unbound RBCs, cells were fixed with 4% paraformaldehyde (PFA) and analyzed using the Zeiss LSM 980 with Airyscan 2. The uptake of RBCs with Mφ was minimal at time point zero, with increasing binding and phagocytosis at the following time points. After 24 hours incubation, macrophages population containing AnxA5-AF555 and/or DiD-labelled RBC-components were observed at the Mφ:RBC ratio 1:100.

The clearance of aged RBCs is mediated by red pulp macrophages in the spleen (54). To model this process *in vitro*, monocyte-derived macrophages were incubated with AnxA5-loaded RBCs at macrophage:RBC ratios of 1:20, 1:50, and 1:100 for 0, 1, 4, and 24 hours (**Figure 3**). RBCs were first labeled with the DiD membrane dye and subsequently treated with PMA, generating a mixed population of DiD-labelled intact RBCs and RBC-derived MVs. Confocal microscopy was used to visualize uptake of the AnxA5-labeled RBC components by macrophages. At time zero, as expected, no internalization of DiD-labeled RBC components or AnxA5 fusion proteins was observed. By 1 hour, DiD signal became detectable within macrophages, indicating the onset of phagocytosis of RBC components. No consistent differences in uptake were observed for different macrophage:RBC ratios, so a ratio of 1:100 was used for all subsequent experiments. Internalization of DiD-labeled RBC components increased over time, with more macrophages exhibiting DiD signal at 4 and 24 hours when compared to earlier time points. The pattern of internalization varied among individual macrophages. Notably, at the 1 hour time point, AnxA5-AF555 was detected in a larger number of macrophages when compared to DiD-labelled RBC components (**Figure 3**). Subsets of macrophages appeared to exclusively internalize AnxA5-AF555, whereas other macrophages contained primarily DiD-labelled RBC components. This difference may reflect distinct uptake kinetics or a possible dissociation of AnxA5 from RBC-derived MVs prior internalization, potentially induced by the relatively low calcium concentration in the IMDM medium used during co-incubation (~1.5 mM CaCl_2_). For comparison, AnxA5 binding to RBC-derived microvesicles increases progressively with calcium, becoming detectable at 1 mM and reaching maximal levels around 5 mM (**Supplementary Figure 2**).

### FVIII-AnxA5 derived peptides presented on HLA-DR

**Figure 4:**
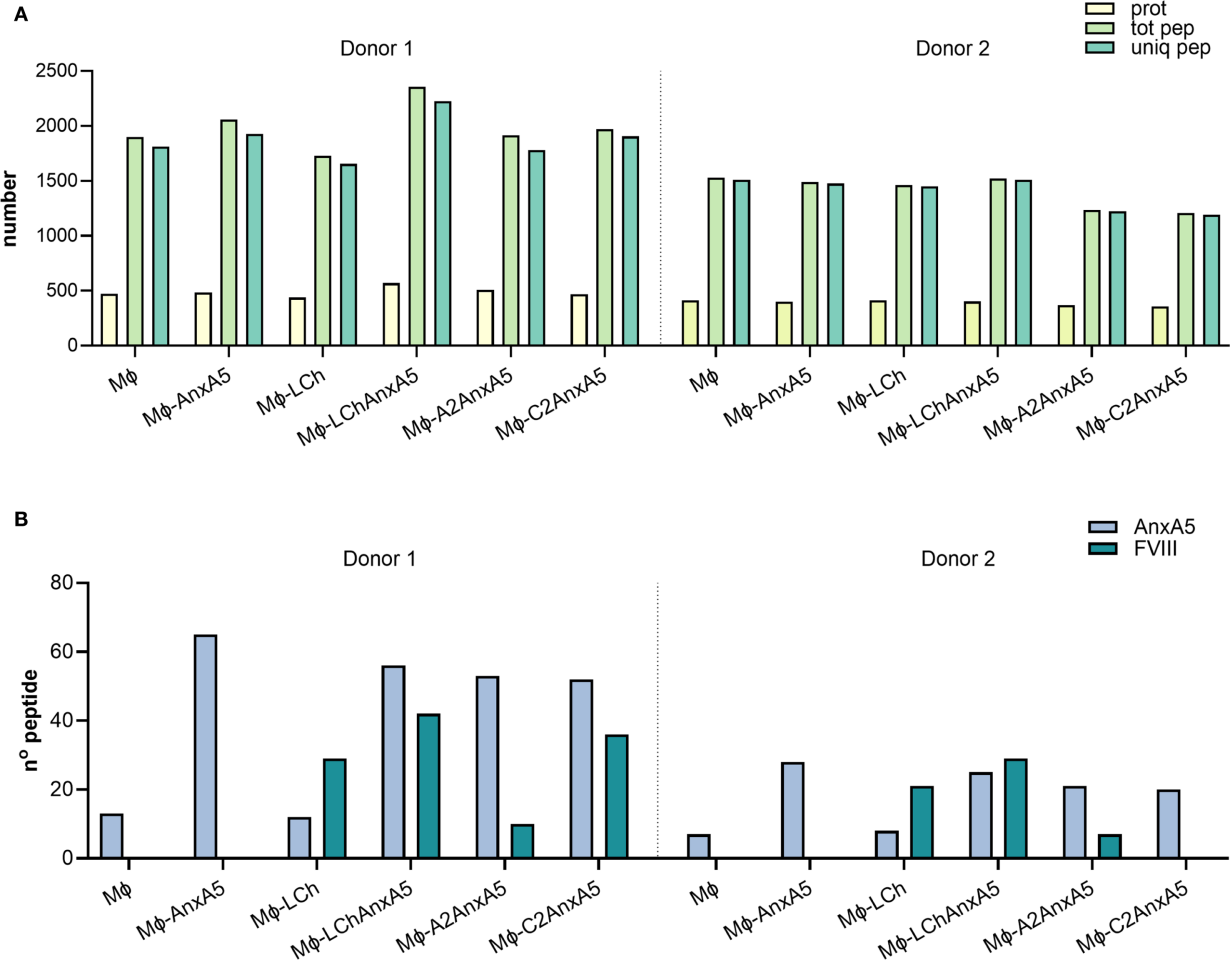
FVIII-Anx5 derived peptides presented on HLA-DR. To evaluate whether FVIII–Anx5 fusion proteins are processed and presented by macrophages, monocyte-derived macrophages from two donors were incubated with the following conditions: untreated (MO), Annexin A5 (MO+Anx5), FVIII light chain (MO+LCh), or FVIII–Anx5 fusion proteins (MO+LChAnx5, MO+A2Anx5, MO+C2Anx5), in the absence of RBCs. Data for the two donors are shown separately, divided by a dashed line. **(A)** Barplot showing the total number of proteins (prot, pale yellow-green), total peptides (tot pep, light green), and unique peptides (uniq pep, medium aquamarine) identified by mass spectrometry for each condition. **(B)** Barplot showing the number of peptides specifically derived from Annexin A5 (Anx5, light steel blue) and FVIII (teal blue). Peptide identification was performed using Proteome Discoverer.

To evaluate the potential of FVIII–AnxA5 fusion proteins to promote antigen presentation, monocyte-derived macrophages from two donors were incubated with purified FVIII-AnxA5 fusion proteins. The experimental conditions included macrophages alone, macrophages incubated with AnxA5, LCh–AnxA5, as well as A2–AnxA5 and C2–AnxA5 fusion proteins; factor VIII light chain (LCh) was included as a control. Upon overnight incubation with 100 nM of the different fusion proteins, macrophages were lysed and HLA-DR peptides complexes were purified using L243 Sepharose. Peptides were eluted from HLA-DR molecules and analyzed by mass spectrometry. Mass spectrometry data were processed using Proteome Discoverer which allowed for identification and quantification of peptides presented by macrophages. The number of identified proteins, peptides, and unique peptides per condition is summarized in the bar plot shown in **Figure 4A** and detailed in **Supplementary Table 1A**. Antigen processing results in multiple MHC class II binding peptides with overlapping sequences, due to progressive trimming at their N- and C-termini (55). This processing step generates peptide with a different length which all possess the core sequence stably bound within the MHC binding groove. Across both donors, an average of 1639 ± 304 unique peptides per condition was identified, indicating relatively low inter-donor variability. Importantly, peptide counts were consistent across conditions within each donor, reflecting robust and reproducible antigen presentation. We next examined whether peptides derived from the fusion proteins could be detected (**Figure 4B**; **Supplementary Table 1A**). Interestingly, AnxA5-derived peptides were already identified in unstimulated macrophages, likely reflecting the processing of endogenous AnxA5. Upon addition of exogenous AnxA5, the number of AnxA5-derived peptides increased substantially in both donors, indicating that the detected peptides originated from exogenously added AnxA5. When macrophages were incubated with LCh alone, the number of AnxA5-derived peptides was similar to the unstimulated condition, as expected. However, FVIII-derived peptides were detected, demonstrating effective presentation of LCh domain derived peptides on HLA-DR (**Figure 4B**; **Supplementary Table 1A**).

Focusing on the fusion proteins containing FVIII domains, AnxA5-derived peptides were consistently detected at similar levels across the LCh–AnxA5, A2–AnxA5, and C2–AnxA5 conditions in both donors, reflecting efficient processing of the AnxA5 domain within these fusion constructs. This consistency allowed us to concentrate on the detection and variability of FVIII-derived peptides (**Figure 4B**; **Supplementary Table 1A**). In macrophages stimulated with fusion proteins containing FVIII domains, detection of FVIII-derived peptides varied by domain and donor. For LCh–AnxA5, fusion to AnxA5 enhanced presentation of LCh-derived peptides compared to LCh alone. Similarly, stimulation with A2–AnxA5 led to consistent detection of peptides derived from the A2 domain. For C2–AnxA5, donor-specific differences emerged: donor 1 presented both C2 domain and AnxA5-derived peptides, whereas donor 2 predominantly displayed AnxA5 peptides with no detectable C2 domain derived peptides (**Figure 4B**; **Supplementary Table 1A**). This pattern corresponds to the reduced presentation of C2 domain derived peptides observed in donor 2 under the LCh–AnxA5 condition. Although HLA typing data were unavailable, these findings likely reflect individual variability in HLA class II-mediated antigen processing and presentation of FVIII-derived peptides. The dataset was re-analyzed using FragPipe to confirm the findings obtained using Proteome Discoverer. This re-analysis resulted in the identification of a higher number of proteins and an average of 2139 ± 388 unique peptides per condition (**Supplementary Table 1A**, **Supplementary Figure 3A**). Importantly, the number and identity of FVIII–AnxA5–derived peptides detected per condition and per donor were consistent with those identified using Proteome Discoverer (**Supplementary Table 1A**, **Supplementary Figure 3B**). The peptide sequences identified for each fusion protein, condition, and donor in both analyses are detailed in **Supplementary Table 2** (AnxA5), **Supplementary Table 3** (LCh–AnxA5), **Supplementary Table 4** (A2–AnxA5), and **Supplementary Table 5** (C2–AnxA5). These results demonstrate that FVIII–AnxA5 fusion proteins are effectively processed and presented by macrophages.

### Peptide presentation on macrophages incubated with FVIII-AnxA5 loaded RBCs

**Figure 5:**
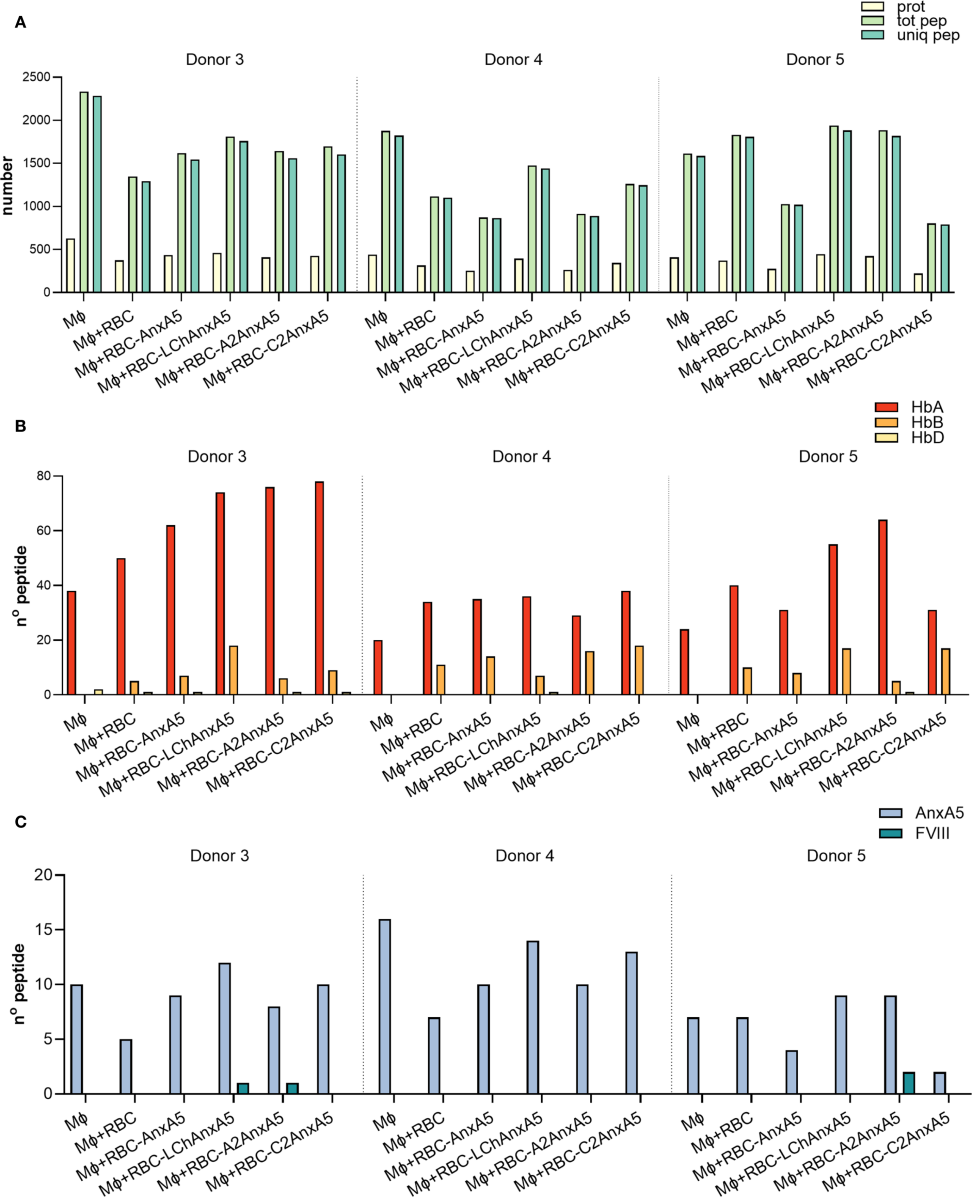
FVIII- AnxA5 loaded RBCs derived peptides presented on HLA-DR. To assess antigen presentation in the context of FVIII–AnxA5–loaded RBCs, monocyte-derived macrophages (Mφ) from three donors were incubated overnight with medium only, untreated RBCs or RBCs pre-loaded with Annexin A5 (AnxA5), LCh– AnxA5, A2– AnxA5, or C2– AnxA5 fusion proteins. Data are presented separately for two donors, divided by a dashed line. **(A)** Barplot showing the total number of proteins (prot, pale yellow-green), total peptides (tot pep, light green), and unique peptides (uniq pep, medium aquamarine) identified by mass spectrometry for each condition. **(B)** Barplot showing the number of peptides derived from hemoglobin alpha (HbA, pale yellow), beta (HbB, orange-yellow), and delta (HbD, vivid red-orange) chains. **(C)** Barplot showing the number of peptides specifically derived from Annexin A5 (Anx5, light steel blue) and FVIII (teal blue). Peptide identification was performed using Proteome Discoverer.

Building on our analysis of macrophages incubated directly with fusion proteins, we next evaluated antigen presentation following phagocytosis of RBCs and RBC-derived MVs. After uptake, macrophages process RBCs and RBC-derived MVs, finally resulting in loading of RBC-derived peptides onto MHC class II. Experimental conditions included macrophages alone, macrophages incubated with PMA stimulated RBCs, and macrophages incubated with PMA stimulated RBCs pre-loaded with AnxA5, LCh–AnxA5, A2–AnxA5, or C2–AnxA5 fusion proteins. Following overnight incubation, peptides were eluted from HLA-DR molecules and analyzed by mass spectrometry. Data were processed using Proteome Discoverer. The number of identified proteins, peptides, and unique peptides per condition is summarized in **Figure 5A** and detailed in **Supplementary Table 1B**. Data from three donors allowed assessment of inter-donor variability. Across all donors, an average of 1462 ± 414 unique peptides were detected. Hemoglobin-derived peptides were identified even in conditions without added RBCs, likely due to hemolysis during PBMC isolation and subsequent uptake of lysed RBCs by monocytes. Importantly, pulsing of macrophages with RBCs resulted in significantly higher number of hemoglobin derived peptide presented on MHC class II (**Figure 5B**; **Supplementary Table 1B**). AnxA5-derived peptides were also detected in control conditions without the addition of FVIII-AnxA5 fusion proteins, consistent with prior findings (**Figure 5C**; **Supplementary Table 1B**). Addition of FVIII–AnxA5 fusion proteins resulted in AnxA5-derived peptide presentation at levels similar to control conditions lacking FVIII-AnxA5 fusion proteins. Detection of FVIII domain peptides varied among donors and fusion constructs: donor 3 presented one peptide each from LCh–AnxA5 and A2–AnxA5; donor 4 had no detectable FVIII peptides; donor 5 showed two peptides from A2–AnxA5 (**Figure 5C**; **Supplementary Table 1B**). To further evaluate these findings, we applied FragPipe to the dataset. This approach revealed a broader proteome coverage, identifying more proteins and an average of 2,059 ± 527 unique peptides per condition, as shown in **Supplementary Figure 4A** and **Supplementary Table 1B**. Despite the higher number of peptides identified, the number of hemoglobin- and AnxA5-derived peptides remained similar to that identified with Proteome Discoverer (**Supplementary Figures 4B, C**). Notably, FVIII-derived peptides were not detected in any condition, with the exception of a single LCh-derived peptide observed in donor 3. Peptide sequences for both analyses are provided in **Supplementary Tables 2**-**5**, where FVIII-derived peptides previously reported as immunogenic are underlined. These results demonstrate that macrophages efficiently process and present RBC-derived peptides. However, FVIII–AnxA5 peptide presentation is markedly less efficient when delivered via RBCs compared to direct loading of fusion proteins onto macrophages.

## Discussion

The development of inhibitors remains a major complication in FVIII replacement therapy for patients with hemophilia A (5). While non-factor replacement therapies such as emicizumab have recently emerged, FVIII administration remains indispensable in certain clinical situations such as acute bleeding or surgical interventions (16). This underscores the continued need for strategies aimed at modulating the immune response to FVIII and promoting antigen-specific tolerance. To this end, we designed fusion proteins combining the highly immunogenic A2, C1, and C2 domains of FVIII with Annexin A5 (AnxA5), a protein with high affinity for phosphatidylserine (PS), resulting in the constructs A2–AnxA5, C2–AnxA5, and A3–C1–C2 light chain–AnxA5 (LCh–AnxA5) (49, 56). PS is externalized on the surface of apoptotic cells, which are typically cleared by phagocytes in a non-inflammatory manner (57). It is well-established that the binding of AnxA5 to PS is calcium-dependent; elegant studies by Andree et al. shown that at 1 mM Ca^2+^ significant amount of AnxA5 can bind to phospholipid surfaces containing 5% of PS (53). In agreement with this, we showed that AnxA5 can bind to RBC-derived MVs in medium containing 1 mM Ca^2+^ (**Supplementary Figure 2**). Binding of the FVIII-AnxA5 fusion proteins to biological PS-containing membranes *in vivo* would be highly dependent on local Ca^2+^ concentrations. Therefore, we expect that FVIII-AnxA5 fusion proteins will not fully cover PS-containing membranes on apoptotic cells.

The FVIII-AnxA5 fusion proteins generated in this study bind highly efficient to immobilized PS. We compared the efficacy of PS binding of FVIII-AnxA5 fusion proteins with that of isolated FVIII light chain (LCh). FVIII LCh binds PS through its carboxy-terminal C1 and C2 domains (50, 51, 58). Our data show that FVIII-AnxA5 fusion proteins bind more efficiently to PS when compared to FVIII LCh. Based on this observation, we expect that FVIII-AnxA5 fusion proteins will readily bind to PS containing cellular membranes *in vivo*. We further demonstrated that fusion proteins are efficiently taken up by macrophages. Mass spectrometry analysis confirmed that macrophages process and present peptides derived from both FVIII and Annexin A5, suggesting successful antigen delivery into the MHC class II pathway. This finding is particularly relevant given that PS-mediated clearance is primarily executed by tolerogenic macrophage subsets, such as red pulp macrophages (RPMs) in the spleen and Kupffer cells (KCs) in the liver (59). These macrophage populations specialize in the non-inflammatory clearance of apoptotic and aged cells, and they are known to promote immune tolerance through secretion of anti-inflammatory cytokines (IL-10, TGF-β) and the induction of regulatory T cells (Tregs) (35, 42). By fusing FVIII to Annexin A5, we label apoptotic cargo and aim to shift FVIII recognition away from immunogenic pathways, such as marginal zone B-cell/cDC-mediated presentation, toward tolerogenic routes dominated by RPMs and KCs (23).

Supporting this hypothesis, previous studies have demonstrated that PS-containing liposomes loaded with recombinant human acid alpha-glucosidase and FVIII can promote tolerance (60, 61). This tolerogenic effect of PS is antigen-specific and involves increased secretion of TGF- β by antigen presenting cells and promotes expansion of regulatory T cells (60, 61). The core domain of AnxA5 appears responsible for tolerogenic effects on dendritic cells, inhibiting pro-inflammatory responses and costimulatory molecule expression (62). Likewise, binding of nanoparticles coated with the AnxA1-core domain to dendritic cells induced antigen-specific immunosuppression by promoting an anergy-like state in CD4^+^ T cells (63). Literature data suggest that AnxA5 itself plays a multifaceted role in modulating immune responses to dying cells. It has been postulated that the binding of AnxA5 to PS exposed on apoptotic and necrotic cells may potentially interfere with their immunosuppressive effects (64). AnxA5 knockout mice show reduced immune reactions against allogeneic necrotic cells and increased anti-inflammatory responses of macrophages, suggesting that endogenous AnxA5 promotes inflammation (65). AnxA5 can also function as an immune checkpoint inhibitor by blocking PS-mediated immunosuppression in the tumor microenvironment, enhancing anti-tumor immunity (66). Together these findings indicate that AnxA5 has context-dependent effects on immunotolerance, potentially acting as both pro-inflammatory and immunosuppressive mediators depending on the specific cellular environment and experimental conditions. Our findings suggest that AnxA5-chimeras may have potential therapeutic applications in modulating immune responses to protein-based therapeutics.

Interestingly, our data show that FVIII-AnxA5 fusion proteins were efficiently processed and presented on MHC class II. Also, RBCs and RBC-derived MVs were efficiently processed by macrophages resulting in presentation of RBC-derived peptides on MHC class II. RBCs loaded with FVIII-AnxA5 fusion proteins were also efficiently internalized by macrophages. Under this experimental condition, a very limited number of FVIII-AnxA5 derived peptides were presented on MHC class II. We hypothesize that the absence of FVIII-derived peptides in the FVIII-AnxA5 loaded RBCs is due to limited surface binding of FVIII-AnxA5 fusion proteins to PS-containing RBC membranes. Alternatively, FVIII-AnxA5 derived peptides may not efficiently compete with abundantly MHC class II presented endogenous or RBC-derived peptides. Follow-up studies are needed to further optimize RBC mediated targeting of FVIII-AnxA5 fusion proteins generated in this study. Notably, several of the FVIII-derived peptides identified correspond to epitopes previously reported as immunogenic, highlighting the potential of FVIII-AnxA5 fusion proteins to ultimately promote antigen-specific tolerance toward clinically relevant FVIII epitopes (67–77).

In conclusion, our studies confirm efficient uptake of FVIII-AnxA5 fusion proteins and MHC class II peptide presentation by macrophages. Our approach provides a potential basis for the design on a platform to mitigate FVIII immunogenicity. Future studies will be needed to clarify whether FVIII–AnxA5–loaded macrophages induce tolerogenic T-cell responses, such as anergy or regulatory T-cell expansion, or instead trigger immune activation, and subsequently validate these immune-modulatory effects *in vivo*. Together, our findings provide an avenue for the development of a PS-targeted antigen delivery system for the induction of tolerance in hemophilia A, and potentially also other antibody mediated disorders.

## Data Availability

The mass spectrometry proteomics data have been deposited to the ProteomeXchange Consortium via the PRIDE [1] partner repository with the dataset identifier PXD068424 [1] Perez-Riverol Y, Bandla C, Kundu DJ, Kamatchinathan S, Baia J, Hewapathirana S, John NS, Prakash A, Walzer M, Wang S, Vizcaíno JA. The PRIDE database at 20 years: 2025 update. Nucleic Acids Res. 2025 Jan 6;53(D1):D543-D553. doi: 10.1093/nar/gkae1011
