# Supplementary material for: MHC class II presentation of FVIII-AnnexinA5 fusion proteins internalized by antigen presenting cells
Source: Front Immunol. 2025 Sep 25;16:1668397. doi: 10.3389/fimmu.2025.1668397 (PMC12507630; doi:10.3389/fimmu.2025.1668397)
Supplement: Supplementary file 1 [file DataSheet1.pdf]

Supplementary Material

Supplementary Figure 1

Anx:

MAQVLRGTVTDFPGFDERADAETLRKAMKGLGTDEESILTLLTSRSNAQRQEISAAFKTLFGRDLLDDLKSELTGKFEKLIVALMKPSRLYDAYELKH
ALKGAGTNEKVLTEIIASRTPEELRAIKQVYEEEYGSSLEDDVVGDTSGYYQRMLVVLLQANRDPDAGIDEAQVEQDAQALFQAGELKWGTDEEKF
ITIFGTRSVSHLRKVFDKYMTISGFQIEETIDRETSGNLEQLLLAVVKSIRSIPAYLAETLYYAMKGAGTDDHTLIRVMVSRSEIDLFNIRKEFRKNFATSL
YSMIKGDTSGDYKKALLLLCGEDDARGHPFEGKPIPNPLLGLDSTRTGWSHPQFEKGGGSGGGSGGSAWSHPQFEK

LCh:

METDTLLLWVLLLWVPGSTGDEITRTTLQSDQEEIDYDDTISVEMKKEDFDIYDEDENQSPRSFQKKTRHYFIAAVERLWDYGMSSSPHVLRNRA
QSGSVPQFKKVVFQEFTDGSFTQPLYRGELNEHLGLLGPYIRAEVEDNIMVTFRNQASRPYSFYSSLISYEEDQRQGAEPRKNFVKPNETKTYFWK
VQHHMAPTKDEFDCKAWAYFSDVDLEKDVHSGLIGPLLVCHTNTLNPAHGRQVTVQEFALFFTIFDETKSWYFTENMERNCRAPCNIQMEDPTF
KENYRFHAINGYIMDTLPGLVMAQDQRIRWYLLSMGSNENIHSIHFSGHVFTVRKKEEYKMALYNLYPGVFETVEMLPSKAGIWRVECLIGEHLH
AGMSTLFLVYSNKCQTPLGMASGHIRDFQITASGQYGQWAPKLARLHYSGSINAWSTKEPFSWIKVDLLAPMIIHGIKTQGARQKFSSLYISQFIIM
YSLDGKKWQTYRGNSTGTLMVFFGNVDSSGIKHNIFNPPIIARYIRLHPTHYSIRSTLRMELMGCDLNSCSMPLGMESKAISDAQITASSYFTNMF
ATWSPSKARLHLQGRSNAWRPQVNNPKEWLQVDFQKTMKVTGVTTQGVKSLLTSMYVKEFLISSSQDGHQWTLFFQNGKVKVFQGNQDSFT
PVVNSLDPPLLTRYLRIHPQSWVHQIALRMEVLGCEAQDLYARGHPFEGKPIPNPLLGLDSTRTGWSHPQFEKGGGSGGGSGGSAWSHPQFEK

A2-Anx5:

SVAKKHPKTWVHYIAAEEEDWDYAPLVLAPDDRSYKSQYLNNGPQRIGRKYKKVRFMAYTDETFKTREAIQHESGILGPLLYGEVGDTLLIIFKNQA
SRPYNIYPHGITDVRPLYSRRLPKGVKHLKDFPILPGEIFKYKWTVTVEDGPTKSDPRCLTRYYSSFVNMERDLASGLIGPLLICYKESVDQRGNQIMS
DKRNVILFSVFDENRSWYLTENIQRFLPNPAGVQLEDPEFQASNIMHSINGYVFDSLQLSVCLHEVAYWYILSIGAQTDFLSVFFSGYTFKHKMVYE
DTLTLFPFSGETVFMSMENPGLWILGCHNSDFRNRGMTALLKVSSCDKNTGDYYEDSYEDISAYLLSKNNAIEPAGGGGSGGGGSGGGGSMAQV
LRGTVTDFPGFDERADAETLRKAMKGLGTDEESILTLLTSRSNAQRQEISAAFKTLFGRDLLDDLKSELTGKFEKLIVALMKPSRLYDAYELKHALKGA
GTNEKVLTEIIASRTPEELRAIKQVYEEEYGSSLEDDVVGDTSGYYQRMLVVLLQANRDPDAGIDEAQVEQDAQALFQAGELKWGTDEEKFITIFGT
RSVSHLRKVFDKYMTISGFQIEETIDRETSGNLEQLLLAVVKSIRSIPAYLAETLYYAMKGAGTDDHTLIRVMVSRSEIDLFNIRKEFRKNFATSLYSMIK
GDTSGDYKKALLLLCGEDDARGHPFEGKPIPNPLLGLDSTRTGWSHPQFEKGGGSGGGSGGSAWSHPQFEK

C2-Anx5:

SCSMPLGMESKAISDAQITASSYFTNMFATWSPSKARLHLQGRSNAWRPQVNNPKEWLQVDFQKTMKVTGVTTQGVKSLLTSMYVKEFLISSSQ
DGHQWTLFFQNGKVKVFQGNQDSFTPVVNSLDPPLLTRYLRIHPQSWVHQIALRMEVLGCEAQDLYGGGGSGGGGSGGGGSMAQVLRGTVT
DFPGFDERADAETLRKAMKGLGTDEESILTLLTSRSNAQRQEISAAFKTLFGRDLLDDLKSELTGKFEKLIVALMKPSRLYDAYELKHALKGAGTNEKV
LTEIIASRTPEELRAIKQVYEEEYGSSLEDDVVGDTSGYYQRMLVVLLQANRDPDAGIDEAQVEQDAQALFQAGELKWGTDEEKFITIFGTRSVSHL
RKVFDKYMTISGFQIEETIDRETSGNLEQLLLAVVKSIRSIPAYLAETLYYAMKGAGTDDHTLIRVMVSRSEIDLFNIRKEFRKNFATSLYSMIKGDTSG
DYKKALLLLCGEDDARGHPFEGKPIPNPLLGLDSTRTGWSHPQFEKGGGSGGGSGGSAWSHPQFEK

LCh-Anx5:

METDTLLLWVLLLWVPGSTGDEITRTTLQSDQEEIDYDDTISVEMKKEDFDIYDEDENQSPRSFQKKTRHYFIAAVERLWDYGMSSSPHVLRNRA
QSGSVPQFKKVVFQEFTDGSFTQPLYRGELNEHLGLLGPYIRAEVEDNIMVTFRNQASRPYSFYSSLISYEEDQRQGAEPRKNFVKPNETKTYFWK
VQHHMAPTKDEFDCKAWAYFSDVDLEKDVHSGLIGPLLVCHTNTLNPAHGRQVTVQEFALFFTIFDETKSWYFTENMERNCRAPCNIQMEDPTF
KENYRFHAINGYIMDTLPGLVMAQDQRIRWYLLSMGSNENIHSIHFSGHVFTVRKKEEYKMALYNLYPGVFETVEMLPSKAGIWRVECLIGEHLH
AGMSTLFLVYSNKCQTPLGMASGHIRDFQITASGQYGQWAPKLARLHYSGSINAWSTKEPFSWIKVDLLAPMIIHGIKTQGARQKFSSLYISQFIIM
YSLDGKKWQTYRGNSTGTLMVFFGNVDSSGIKHNIFNPPIIARYIRLHPTHYSIRSTLRMELMGCDLNSCSMPLGMESKAISDAQITASSYFTNMF
ATWSPSKARLHLQGRSNAWRPQVNNPKEWLQVDFQKTMKVTGVTTQGVKSLLTSMYVKEFLISSSQDGHQWTLFFQNGKVKVFQGNQDSFT
PVVNSLDPPLLTRYLRIHPQSWVHQIALRMEVLGCEAQDLYGGGGSGGGGSGGGGSMAQVLRGTVTDFPGFDERADAETLRKAMKGLGTDEES
ILTLLTSRSNAQRQEISAAFKTLFGRDLLDDLKSELTGKFEKLIVALMKPSRLYDAYELKHALKGAGTNEKVLTEIIASRTPEELRAIKQVYEEEYGSSLED
DVVGDTSGYYQRMLVVLLQANRDPDAGIDEAQVEQDAQALFQAGELKWGTDEEKFITIFGTRSVSHLRKVFDKYMTISGFQIEETIDRETSGNLE
QLLLAVVKSIRSIPAYLAETLYYAMKGAGTDDHTLIRVMVSRSEIDLFNIRKEFRKNFATSLYSMIKGDTSGDYKKALLLLCGEDDARGHPFEGKPIPNP
LLGLDSTRTGWSHPQFEKGGGSGGGSGGSAWSHPQFEK

Supplementary Figure 2

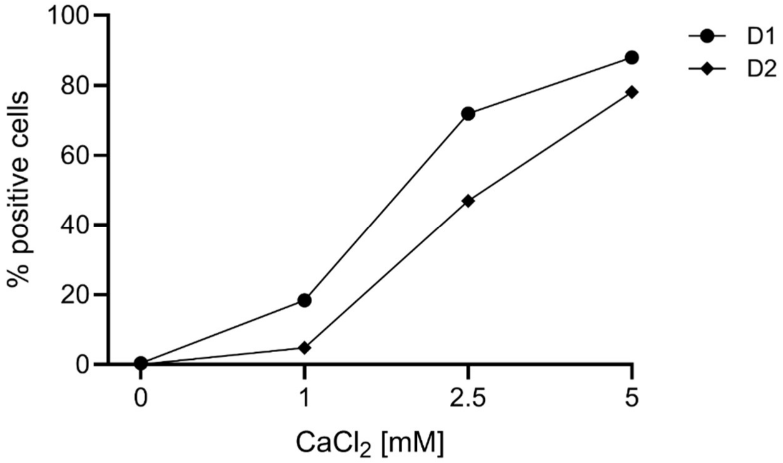

Supplementary Figure 3

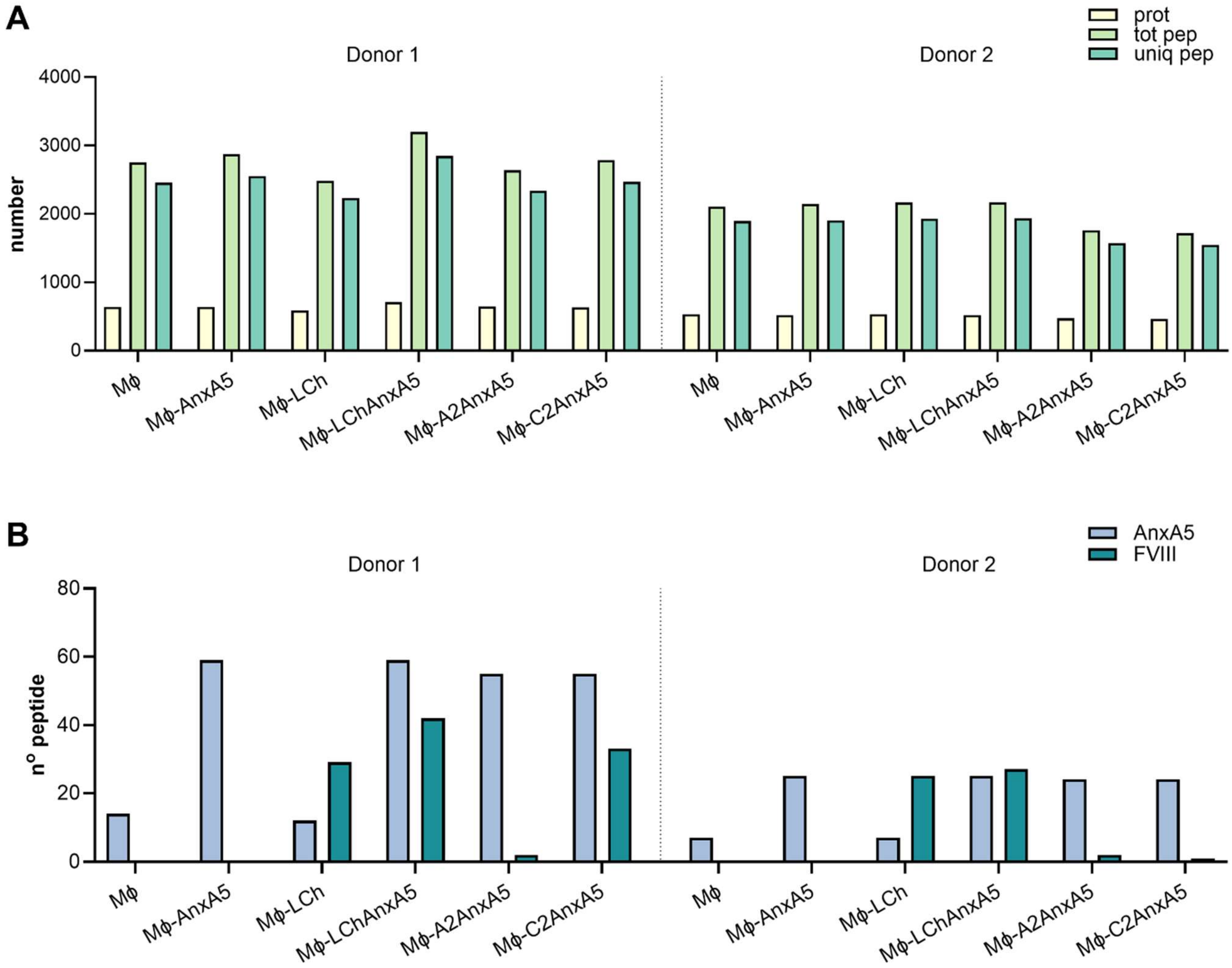

Supplementary Figure 4

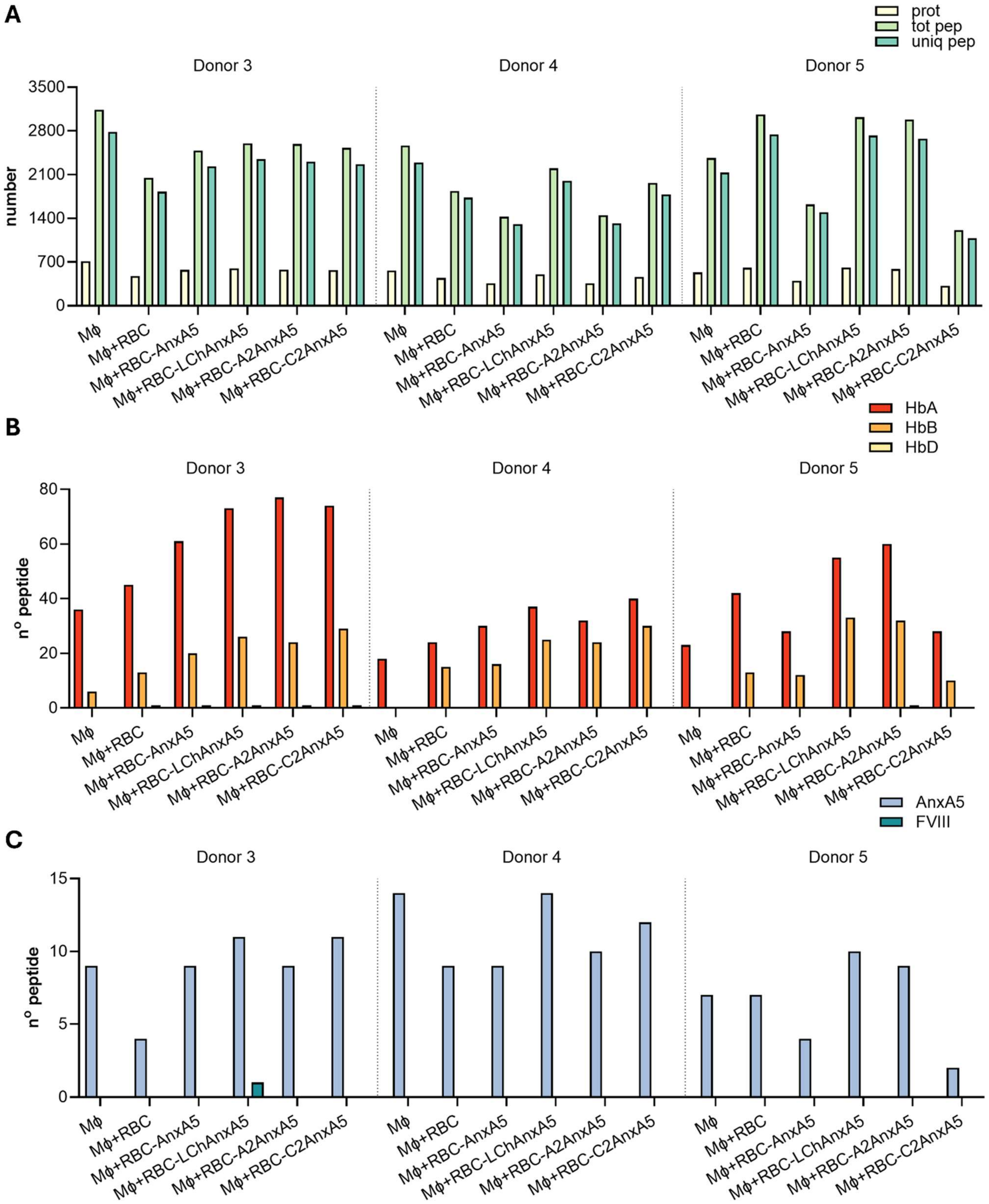

References

1. Porcheddu V, Lhomme G, Giraudet R, Correia E, Maillère B. The self-reactive FVIII T cell repertoire in healthy individuals relies on a short set of epitopes and public clonotypes. *Front Immunol* (2024) 15: doi: 10.3389/fimmu.2024.1345195

2. Steinitz KN, van Helden PM, Binder B, Wraith DC, Unterthurner S, Hermann C, et al. CD4+ T-cell epitopes associated with antibody responses after intravenously and subcutaneously applied human FVIII in humanized hemophilic E17 HLA-DRB1*1501 mice. *Blood* (2012) 119:4073–4082. doi: 10.1182/blood-2011-08-374645

3. Reding MT, Okita DK, Diethelm-Okita BM, Anderson TA, Conti-Fine BM. Epitope repertoire of human CD4 T cells on the A3 domain of coagulation factor VIII. *Journal of Thrombosis and Haemostasis* (2004) 2:1385–1394. doi: 10.1111/j.1538-7836.2004.00850.x

4. Ettinger RA, Paz P, James EA, Gunasekera D, Aswad F, Thompson AR, et al. T cells from hemophilia A subjects recognize the same HLA-restricted FVIII epitope with a narrow TCR repertoire. *Blood* (2016) 128:2043–2054. doi: 10.1182/blood-2015-11-682468

5. Jones TD, Phillips WJ, Smith BJ, Bamford CA, Nayee PD, Baglin TP, et al. Identification and removal of a promiscuous CD4+ T cell epitope from the C1 domain of factor VIII. *Journal of Thrombosis and Haemostasis* (2005) 3:991–1000. doi: 10.1111/j.1538-7836.2005.01309.x

6. Ettinger RA, James EA, Kwok WW, Thompson AR, Pratt KP. HLA-DR-restricted T-cell responses to factor VIII epitopes in a mild haemophilia A family with missense substitution A2201P. *Haemophilia* (2010) 16:44–55. doi: 10.1111/j.1365-2516.2008.01905.x

7. Gunasekera D, Vir P, Karim AF, Ragni M V., Pratt KP. Hemophilia A subjects with an intron-22 gene inversion mutation show CD4+ T-effector responses to multiple epitopes in FVIII. *Front Immunol* (2023) 14: doi: 10.3389/fimmu.2023.1128641

8. James EA, Kwok WW, Ettinger RA, Thompson AR, Pratt KP. T-cell responses over time in a mild hemophilia A inhibitor subject: epitope identification and transient immunogenicity of the corresponding self-peptide. *Journal of Thrombosis and Haemostasis* (2007) 5:2399–2407. doi: 10.1111/j.1538-7836.2007.02762.x

9. Ettinger RA, James EA, Kwok WW, Thompson AR, Pratt KP. Lineages of human T-cell clones, including T helper 17/T helper 1 cells, isolated at different stages of anti–factor VIII immune responses. *Blood* (2009) 114:1423–1428. doi: 10.1182/blood-2009-01-200725

10. Ettinger RA, Liberman JA, Gunasekera D, Puranik K, James EA, Thompson AR, et al. FVIII proteins with a modified immunodominant T-cell epitope exhibit reduced immunogenicity and normal FVIII activity. *Blood Adv* (2018) 2:309–322. doi: 10.1182/bloodadvances.2017013482

11. Garnier A, Hamieh M, Drouet A, Leprince J, Vivien D, Frébourg T, et al. Artificial antigen-presenting cells expressing HLA class II molecules as an effective tool for amplifying human specific memory CD4^{+} T cells. *Immunol Cell Biol* (2016) 94:662–672. doi: 10.1038/icb.2016.25

Supplementary figure legend:

Supplementary Figure 1. Amino acid sequences of the designed FVIII-AnxA5 fusion proteins. The A2, C2, and light chain (LCh) domains of FVIII (blue) were fused to annexin A5 (AnxA5, green) via a flexible linker (orange). A C-terminal V5 tag and Twin-Strep tag (black) were introduced for detection and purification purposes.

Supplementary Figure 2. Calcium-dependent binding of AnxA5 fusion proteins to RBC-derived microvesicles. RBCs were treated with PMA to induce MV formation and incubated with fluorescent AnxA5 in the presence of increasing concentrations of $CaCl_2$ (0–5 mM). The percentage of AnxA5-positive RBC-derived MVs was quantified by flow cytometry. A calcium-dependent increase in binding was observed for both donors (D1 and D2), with detectable binding from 1 mM and a progressive increase up to 5 mM, confirming the calcium-dependent binding dynamics of AnxA5 to exposed phosphatidylserine.

Supplementary Figure 3: **FVIII-AnxA5 derived peptides presented on HLA-DR identified using FragPipe.** To further characterize FVIII-AnxA5 presentation, HLA-DR–associated peptides previously identified using Proteome Discoverer in figure 4 were reanalyzed using FragPipe. (**A**) Barplot showing the total number of proteins (prot, pale yellow-green), total peptides (tot pep, light green), and unique peptides (uniq pep, medium aquamarine) identified by mass spectrometry for each condition. (**B**) Barplot showing the number of peptides specifically derived from Annexin A5 (Anx5, light steel blue) and FVIII (teal blue).

Supplementary Figure 4: FVIII-Anx5 loaded RBCs derived peptides presented on HLA-DR analyzed using FragPipe. To further characterize antigen presentation in the context of FVIII–Anx5–loaded RBCs, HLA-DR–associated peptides previously identified using Proteome Discoverer in figure 5 were reanalyzed using FragPipe. (**A**) Barplot showing the total number of proteins (prot, pale yellow-green), total peptides (tot pep, light green), and unique peptides (uniq pep, medium aquamarine) identified by mass spectrometry for each condition. (**B**) Barplot showing the number of peptides derived from hemoglobin alpha (HbA, pale yellow), beta (HbB, orange-yellow), and delta (HbD, vivid red-orange) chains. (**C**) Barplot showing the number of peptides specifically derived from Annexin A5 (AnxA5, light steel blue) and FVIII (teal blue).

Supplementary Table 1: Peptides presentation on HLA-DR. Monocyte-derived macrophages from five donors were incubated under different conditions to assess HLA-DR–restricted antigen presentation. Macrophages from Donors 1 and 2 were treated with medium only, Annexin A5 (AnxA5), FVIII light chain (LCh), or FVIII–AnxA5 fusion proteins (LCh–AnxA5, A2–AnxA5, and C2–AnxA5). Macrophages from Donors 3, 4, and 5 were treated with medium only, untreated RBCs, or RBCs pre-loaded with AnxA5, LCh–AnxA5, A2–AnxA5, or C2–AnxA5 fusion proteins. Data were analyzed using both Proteome Discoverer and FragPipe. **Table A** summarizes data from Donors 1 and 2, including the number of proteins (prot), total peptides (tot pep), unique peptides (uniq pep), Annexin A5 peptides (AnxA5 pep), and FVIII peptides (FVIII pep). **Table B** summarizes data from Donors 3, 4, and 5, including proteins (prot), total peptides (tot pep), unique peptides (uniq pep), hemoglobin alpha, beta, and delta peptides (HbA pep, HbB pep, HbD pep), Annexin A5 peptides (AnxA5 pep), and FVIII peptides (FVIII pep).

Supplementary Table 2. Annexin A5–derived peptides presented on HLA-DR. Annexin A5 (AnxA5)–derived peptides presented on HLA-DR molecules were identified across different donors (D1–D5) and experimental conditions: untreated macrophages (Mφ), macrophages with RBCs (Mφ+RBC), AnxA5 alone (Mφ+AnxA5), or RBCs pre-loaded with AnxA5 (Mφ+RBC+AnxA5). For each peptide, the corresponding domain, starting position, and amino acid sequence are reported. Peptides identified for a specific condition and donor are shown in dusty blue if detected only with Proteome Discoverer, in pale lilac if detected only with FragPipe, and in deep cyan if detected with both tools.

Supplementary Table 3. LCh-Annexin A5–derived peptides presented on HLA-DR. LCh-Annexin A5 (AnxA5)–derived peptides presented on HLA-DR molecules were identified across different donors (D1–D5) and experimental conditions: macrophages with LCh only (Mφ+LCh), LCh-AnxA5 (Mφ+LCh-AnxA5), or RBCs pre-loaded with LCh-AnxA5 (Mφ+RBC+LCh-AnxA5). For each peptide, the corresponding domain, starting position, and amino acid sequence are reported. Peptides identified for a specific condition and donor are shown in dusty blue if detected only with Proteome Discoverer, in pale lilac if detected only with FragPipe, and in deep cyan if detected with both tools. Immunogenic FVIII peptides are underlined (1–11).

Supplementary Table 4. A2-Annexin A5–derived peptides presented on HLA-DR. A2-Annexin A5 (AnxA5)–derived peptides presented on HLA-DR molecules were identified across different donors (D1–D5) and experimental conditions: macrophages with A2-AnxA5 (Mφ+A2-AnxA5) or RBCs pre-loaded with A2-AnxA5 (Mφ+RBC+A2-AnxA5). For each peptide, the corresponding domain, starting position, and amino acid sequence are reported. Peptides identified for a specific condition and donor are shown in dusty blue if detected only with Proteome Discoverer, in pale lilac if detected only with FragPipe, and in deep cyan if detected with both tools. Immunogenic FVIII peptides are underlined (2).

Supplementary Table 5. C2-Annexin A5–derived peptides presented on HLA-DR. C2-Annexin A5 (AnxA5)–derived peptides presented on HLA-DR molecules were identified across different donors (D1–D5) and experimental conditions: macrophages with C2-AnxA5 (Mφ+C2-AnxA5) or RBCs pre-loaded with C2-AnxA5 (Mφ+RBC+C2-AnxA5). For each peptide, the corresponding domain, starting position, and amino acid sequence are reported. Peptides identified for a specific condition and donor are shown in dusty blue if detected only with Proteome Discoverer, in pale lilac if detected only with FragPipe, and in deep cyan if detected with both tools. Immunogenic FVIII peptides are underlined (1–11).
